# A Bayesian approach to estimating the population prevalence of mood and anxiety disorders using multiple measures

**DOI:** 10.1017/S2045796020001080

**Published:** 2021-01-08

**Authors:** Jordan Edwards, A. Demetri Pananos, Amardeep Thind, Saverio Stranges, Maria Chiu, Kelly K. Anderson

**Affiliations:** 1 Department of Epidemiology & Biostatistics, The University of Western Ontario, London, Ontario, Canada; 2 Lawson Health Research Institute, London, Ontario, Canada; 3 Interfaculty Program in Public Health, The University of Western Ontario, London, Ontario, Canada; 4 Department of Family Medicine, Schulich School of Medicine & Dentistry, The University of Western Ontario, London, Ontario, Canada; 5 Department of Population Health, Luxembourg Institute of Health, Strassen, Luxembourg; 6 ICES, Toronto, Ontario, Canada; 7 Institute of Health Policy, Management and Evaluation, University of Toronto, Toronto, Ontario, Canada; 8 Department of Psychiatry, The University of Western Ontario, London, Ontario, Canada

**Keywords:** Common mental disorders, research design and methods, epidemiology, diagnosis and classification, Bayesian analysis, prevalence

## Abstract

**Aims:**

There is currently no universally accepted measure for population-based surveillance of mood and anxiety disorders. As such, the use of multiple linked measures could provide a more accurate estimate of population prevalence. Our primary objective was to apply Bayesian methods to two commonly employed population measures of mood and anxiety disorders to make inferences regarding the population prevalence and measurement properties of a combined measure.

**Methods:**

We used data from the 2012 Canadian Community Health Survey – Mental Health linked to health administrative databases in Ontario, Canada. Structured interview diagnoses were obtained from the survey, and health administrative diagnoses were identified using a standardised algorithm. These two prevalence estimates, in addition to data on the concordance between these measures and prior estimates of their psychometric properties, were used to inform our combined estimate. The marginal posterior densities of all parameters were estimated using Hamiltonian Monte Carlo (HMC), a Markov Chain Monte Carlo technique. Summaries of posterior distributions, including the means and 95% equally tailed posterior credible intervals, were used for interpretation of the results.

**Results:**

The combined prevalence mean was 8.6%, with a credible interval of 6.8–10.6%. This combined estimate sits between Bayesian-derived prevalence estimates from administrative data-derived diagnoses (mean = 7.4%) and the survey-derived diagnoses (mean = 13.9%). The results of our sensitivity analysis suggest that varying the specificity of the survey-derived measure has an appreciable impact on the combined posterior prevalence estimate. Our combined posterior prevalence estimate remained stable when varying other prior information. We detected no problematic HMC behaviour, and our posterior predictive checks suggest that our model can reliably recreate our data.

**Conclusions:**

Accurate population-based estimates of disease are the cornerstone of health service planning and resource allocation. As a greater number of linked population data sources become available, so too does the opportunity for researchers to fully capitalise on the data. The true population prevalence of mood and anxiety disorders may reside between estimates obtained from survey data and health administrative data. We have demonstrated how the use of Bayesian approaches may provide a more informed and accurate estimate of mood and anxiety disorders in the population. This work provides a blueprint for future population-based estimates of disease using linked health data.

## Introduction

When it comes to population-based estimates of disease frequency, individual point estimates with confidence intervals are regularly used to inform research and policy. The accuracy of these individual estimates is a product of the strengths and limitations of both the measures and samples used. Theoretically, a more informative population estimate would incorporate prior information on measurement properties and would leverage the strengths of multiple measures to increase accuracy and precision. This integration of multiple sources of data could be useful in improving estimates for population surveillance and research. A good example is the measurement of common mental disorders, such as depression and anxiety, which are among the leading contributors of global morbidity (Walker *et al*., 2015). Accurate, population-based estimates of these disorders are important for our understanding of disease burden and for health service planning and resource allocation (Kirkbride, 2015).

Currently, Bayesian methodology is being used in the estimation of the global burden of disease (James *et al*., 2017). In Canada, the use of Bayesian methodology to estimate the prevalence of schizophrenia has previously been proposed, but has not yet been implemented (Laliberté *et al*., 2015). There are two aspects of a Bayesian analysis that can be used to estimate uncertainty and improve the accuracy of population estimates of the frequency of mood and anxiety disorders. The first is to use prior information from existing studies – for example, evidence from validation studies – which provide the psychometric properties of specific measures of mood and anxiety disorders. These psychometric properties can be used to inform the prevalence and uncertainty surrounding the estimates of the proportion of people meeting the criteria for a clinical diagnosis in the population (Edwards *et al*., 2019*a*, 2019*b*). The second approach is to integrate the results of multiple population-based measures of common mental disorders into one estimate. Two ways that we estimate the prevalence of common mental disorders is the use of structured interview data from surveys (i.e. survey-derived diagnoses) and fee-for-service billing codes from health administrative databases (i.e. administrative-derived diagnoses).

Both of these sources of data provide distinctive population estimates; specifically, a survey-derived community prevalence that includes people identified from a representative population sample, and an administrative-derived prevalence that includes people receiving a clinical diagnosis across the entire population, in places where there are universal health care systems (Sayal *et al*., 2018). These estimates are influenced by the characteristics of the respective sources of data (Furukawa *et al*., 2003; Gary, 2005; Quan *et al*., 2006; Kisely *et al*., 2009; Gulliver *et al*., 2010; Kessler *et al*., 2010; Puyat *et al*., 2013). Generally, surveys offer standardised measures with more limited coverage of the population, whereas administrative data have greater coverage of the population with less depth of information (Drapeau *et al*., 2011; Puyat *et al*., 2013). Previous work suggests that the use of either of these measures alone may identify a selected subgroup of people with a mood or anxiety disorder in the population, thus leading to an over- or underestimation of the true prevalence (Edwards *et al*., 2019*a*, 2019*b*).

To overcome the limitations of using either one of these measures in isolation, the integration of multiple measures can be accomplished using a Bayesian analysis. This allows for inferences on the prevalence and measurement properties of a combined estimate using two or more population-based measures (Joseph *et al*., 1995; Laliberté *et al*., 2015). Our recent work estimating the concordance between survey- and administrative-derived diagnoses of mood or anxiety disorders using a linkage between national survey and provincial health administrative data provides a platform for this analysis (Edwards *et al*., 2019*a*, 2019*b*).

Our objective was to use a Bayesian approach to derive a more informative estimate of the population prevalence of mood and anxiety disorders in Ontario, Canada. By using primary data from an analysis assessing the concordance of two population measures of mood and anxiety disorders (Edwards *et al*., 2019*a*, 2019*b*), along with prior estimates of the measurement properties of the two measures (Haro *et al*., 2006; Doktorchik *et al*., 2019), we may be able to produce a more informed estimate of population prevalence.

## Methods

### Sample and source of data

Our sample was based on the respondents to the Ontario portion of a national population health survey, the 2012 Canadian Community Health Survey – Mental Health (CCHS-MH). This cross-sectional survey collects information on people's health status, health care utilisation, as well as factors related to the determinants of health, and data collection is done via a telephone or in-person interview with staff from Statistics Canada. The respondents to this survey were individually linked to health administrative databases at ICES (formerly known as the Institute for Clinical Evaluative Sciences), which holds all health administrative data from the Ontario Health Insurance Plan (OHIP) and covers nearly the entire population of Ontario (>96%) (Edwards *et al*., 2019*a*, 2019*b*). ICES houses provincial data on inpatient hospitalisations, outpatient physician visits (including primary care) and emergency department visits. The use of data in this project was authorised under Section 45 of Ontario's Personal Health Information Protection Act, which does not require review by a Research Ethics Board.

### Outcome measures

#### Survey-derived diagnoses

World Mental Health – Composite International Diagnostic Interview 3.0 (WHO-CIDI). This standardised instrument assesses mental disorders and conditions according to DSM-IV (Diagnostic and Statistical Manual of Mental Disorders, Fourth Edition) criteria. We used the 12-month measures of depression, bipolar disorder and generalised anxiety disorders, which are derived from questions regarding symptoms of these disorders (Kessler *et al*., 2004; Gilmour, 2014).

#### Administrative-derived diagnoses

We obtained billing data on mood and anxiety disorders from the linked health administrative data using a standardised algorithm, which was similar to a validated algorithm used to identify depressive disorders in other Canadian settings (Alaghehbandan *et al*., 2012; Doktorchik *et al*., 2019). Cases were identified as people with either: (1) hospitalisation for a mood or anxiety disorder; or (2) a visit to a psychiatrist for a mood or anxiety disorder; or (3) at least two physician billing claims (including primary care physicians) or emergency department visits for a mood or anxiety disorder within any 24-month period. Additionally, cases must have had at least one diagnosis code for a mood or anxiety disorder within the 12-month period prior to completing the survey to ensure that the observation period was aligned for survey- and administrative-derived diagnoses. We used a 5-year lookback period prior to completion of the survey to identify cases.

### Psychometric properties

We used prior estimates of the psychometric properties of both measures, which included a validation of the WHO-CIDI structured interview tool compared to the Structural Clinical Interview for DSM (SCID) (Haro *et al*., 2006), as well as a validation of provincial health administrative billing data using electronic medical records and medical chart review (Doktorchik *et al*., 2019). Both of these validation studies assessed the psychometric properties of the measurement of depressive disorders. The survey-derived diagnoses had a sensitivity of 55.3%, a specificity of 93.7%, a positive predictive value of 73.7% and a negative predictive value of 86.8% (Haro *et al*., 2006). Evidence suggests that the psychometric properties for survey-derived diagnoses of anxiety disorder are similar to depressive disorders (sensitivity 54.4%, specificity 90.7%, positive predictive value 74.5%, negative predictive value 80%) (Haro *et al*., 2006). The administrative-derived diagnoses had a sensitivity of 62.9%, a specificity of 93.8%, a positive predictive value of 68.3% and a negative predictive value of 92.3% (see Table 1) (Doktorchik *et al*., 2019). We did not find a validation of administrative-derived diagnoses of anxiety disorders as a comparison, hence we performed a sensitivity analysis to explore the impact of varying psychometric properties on our combined estimate.

**Table 1.** Concordance between survey structured interview and administrative data diagnosed mood and anxiety disorders in Ontario, Canada (Edwards *et al*., 2019a)

	(+) Admin-derived diagnosed	(−) Admin-derived diagnosis	
(+) Survey-derived diagnosis	164 (3.9%)	415 (9.9%)	579 (13.9%)
(–) Survey-derived diagnosis	268 (6.4%)	3310 (79.6%)	3578 (86.1%)
	432 (10.4%)	3725 (89.6%)	4157 (100.0%)

#### Data analysis

Prior estimates of the prevalence, concordance and psychometric properties of mood and anxiety disorders using multiple measures have provided us the opportunity to apply a Bayesian analytic approach. This flexible approach uses prior information from two population measures to inform the conditional probability of a combined prevalence estimate (Joseph *et al*., 1995). A similar approach has been described in detail in a previous publication (Joseph *et al*., 1995). An alternative frequentist approach to this Bayesian analysis would be a meta-analysis, which would not have been able to integrate the concordance information between both measures.

We estimated the posterior densities of all parameters using a Hamiltonian Monte Carlo (HMC), which is a Markov Chain Monte Carlo technique (Neal, 1996; Hoffman and Gelman, 2014). HCM is used to generate random samples from the posterior densities of each parameter, which in turn can be used to compute expectations, quantiles and Bayesian credible intervals. It is preferred over the Gibbs sample, originally used by Joseph *et al*. (1995), as it does not require *β* priors and allows us to specify arbitrary priors which best represent existing knowledge. Priors were selected by using the asymptotic sampling distribution for each statistic, as described in previous studies (Haro *et al*., 2006; Doktorchik *et al*., 2019). Summaries of posterior distributions, including the means and 95% equally tailed posterior credible intervals (95% CI), were used for interpretation of the results. The posterior means are used to estimate the peak of the sampling distribution and can be interpreted as a frequentist prevalence. Credible intervals are Bayesian analogues to 95% confidence intervals. To assess model fit and performance, we assessed diagnostics using Stan, and performed posterior predictive checks using simulated data (Carpenter *et al*., 2017). Twelve chains were used to sample 2000 samples per chain (1000 warmup, 1000 post warmup). All analyses were conducted using R (R Core Team, 2013). The script used for this project is available in online Supplementary material (Appendix 1 available at https://github.com/Dpananos/bayes_multiple_measures).

### Sensitivity analyses

To assess how misspecification of our priors would impact the results, we performed sensitivity analyses that altered the means of our prior distributions for the sensitivities and specificities of both the survey-derived and administrative-derived measures, while holding the variances constant. We varied the prior sensitivities and specificities to 5% smaller and 5% larger than the values we used in our final model (Haro *et al*., 2006; Doktorchik *et al*., 2019).

## Results

The total Ontario sample completing the 2012 CCHS-MH was 5492 people, of whom 1335 (24%) were unable to be linked (~9%) or were unwilling to share their information (~15%) for data linkage (Statistics Canada, 2013). As such, our linked sample included 4157 people, comprised of 1943 men (46.7%) and 2214 women (53.3%). The mean age of the sample was 48.0 (s.d. = 20.1) years. Using a frequentist approach, the survey-derived prevalence from our sample was 13.9% (95% CI 12.8–14.9%), the administrative-derived prevalence was 10.4% (95% CI 9.5–11.3%), and the concordance between the two measures was 19.4%, which has been reported previously (Edwards *et al*., 2019*a*, 2019*b*).

**Fig. 1.**
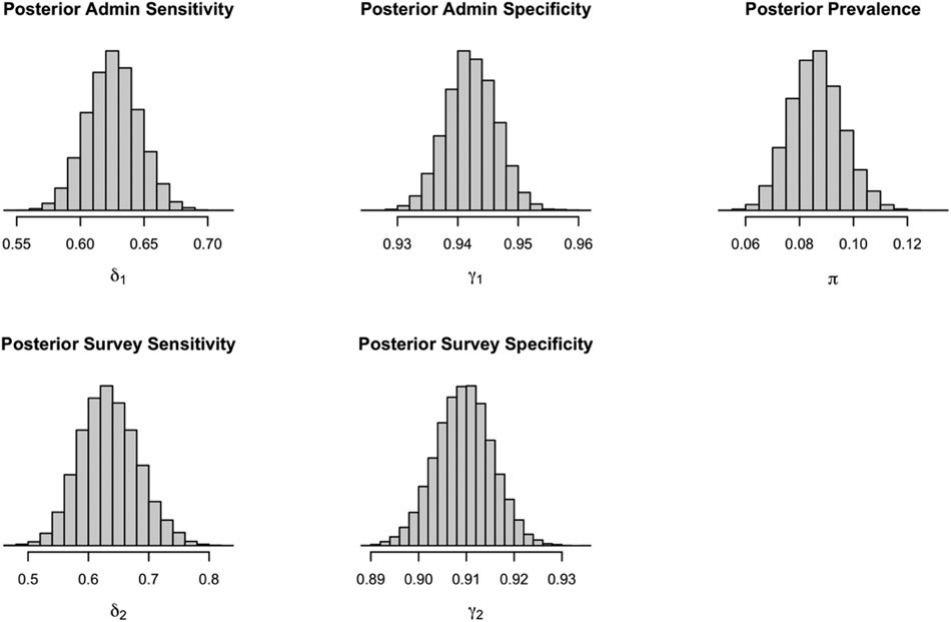
Marginal posterior density for the prevalence of mood or anxiety disorders in Ontario, Canada, using data from both survey and administrative data combined. *Note*: *π* represents posterior prevalence using both administrative and survey data, *δ*_1_ represents sensitivity for administrative data, and *γ*_1_ represents specificity for administrative data, *δ*_2_ represents sensitivity for survey data, and *γ*_2_ represents specificity for survey data.

The results of the Bayesian analysis suggest that the combined prevalence mean was 8.6% with a credible interval of 6.8–10.6% (see Fig. 1, Table 1). This combined estimate sits between our prior informed estimates from administrative-derived diagnoses (mean 7.4%, 95% CI 5.4–9.6%) and the survey-derived diagnoses (mean 13.9%, 95% CI 1.2–25.0%). In our results, the mean estimates were similar to the posterior medians. These estimates differ from the prior prevalence estimate used to inform the models that were derived using a frequentist approach. The large difference in the sample size of the prior validation studies for the psychometric properties of the administrative-derived (*n* = 3362) and our survey-derived (*n* = 325) estimates contributed to the wider posterior distribution for the prior informed survey estimate. The findings in Fig. 1 suggest that results from administrative data alone may be providing an underestimate of the true population prevalence of mood and anxiety disorders, whereas estimates from surveys may be overestimating the population prevalence.

Additionally, the posterior distribution of our combined estimate suggests that administrative-derived estimates have a similar sensitivity (95% CI 59–67%) compared to the survey-derived estimates (95% CI 55–73%). Furthermore, there is high specificity for both administrative- (95% CI 93–95%) and survey-derived (95% CI 89–92%) estimates (see Table 1). The survey-derived estimates have a higher sensitivity than the administrative-derived estimates, though the results of our posterior distribution suggest administrative-derived estimates may have a higher specificity than survey-derived estimates (Table 2).

**Table 2.** Marginal prior and posterior medians and 95% CI of the posterior equally tailed 95% CI for the prevalence (*π*) and sensitivities (*δ*_1_, *δ*_2_) and specificities (*γ*_1_, *γ*_2_) for each measure of mood and anxiety disorder and the combination of the two measures

	Prior information		Admin-derived diagnosis		Survey-derived diagnosis		Both measures	
	Mean	95% CI	Mean	95% CI	Mean	95% CI	Mean	95% CI
*π*			7.4	5.4–9.6	13.9	1.2–25.0	8.6	6.8–10.6
Admin-derived								
*δ*_1_	62.9	59.9–66.8	62.9	58.8–66.8			62.6	58.6–66.6
*γ*_1_	93.8	92.8–94.7	93.8	93.0–94.6			94.2	93.4–95.0
Survey-derived								
*δ*_2_	55.3	41.9–68.6^a^			55.2	51.3–59.1	63.5	54.6–73.4
*γ*_2_	93.7	89.9–97.4^a^			93.0	86.5–99.4	91.0	89.8–92.1

*Note*: *π* represents posterior prevalence, *δ*_1_ represents sensitivity for administrative data, and *γ*_1_ represents specificity for administrative data, *δ*_2_ represents sensitivity for survey data, and *γ*_2_ represents specificity for survey data.

a Estimated from (se) (Higgins, 2008).

**Fig. 2.**
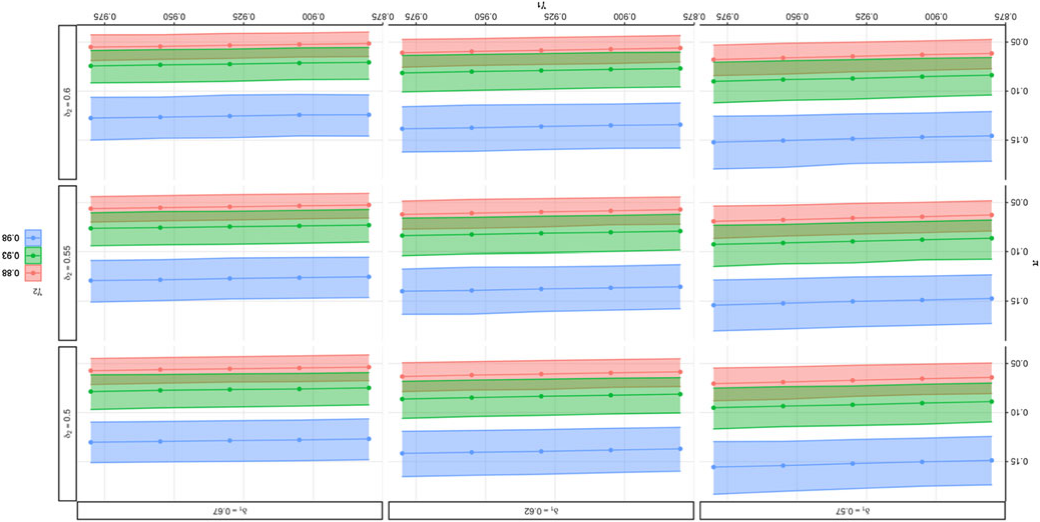
Results from the sensitivity analysis testing the impact of variation in psychometric properties on the posterior prevalence. *Note*: *π* represents posterior prevalence using both administrative and survey data, *δ*_1_ represents sensitivity for administrative data, and *γ*_1_ represents specificity for administrative data, *δ*_2_ represents sensitivity for survey data, and *γ*_2_ represents specificity for survey data. We find that changes in the prior expectation for the sensitivities of both survey and administrative data, as well as the specificity of the administrative data, do not appreciably change the expected prevalence. We do find that changes to the specificity of the survey data have a considerable influence on the expected prevalence. The coloured intervals represent the credible intervals of the expected prevalence with three different values of the specificity for the survey data. Red represents a prior expectation for the specificity of 88%, green 93% and blue 98%.

The results of our sensitivity analyses suggest that changes to the means of the prior psychometric properties of our administrative-derived measure do not modify our combined prevalence estimate in any significant way. Our sensitivity analysis does suggest, however, that while changes in the sensitivity of our survey-derived measure do not appreciably change our combined posterior prevalence estimate, changes in the specificity of the survey-derived measure highlighted by coloured lines in Fig. 2 have an appreciable impact on the combined posterior prevalence estimate. Specifically, when the mean of the posterior specificity is increased from 88 to 98%, there is roughly a 7.5% increase in the combined posterior prevalence estimate (see Fig. 2).

**Fig. 3.**
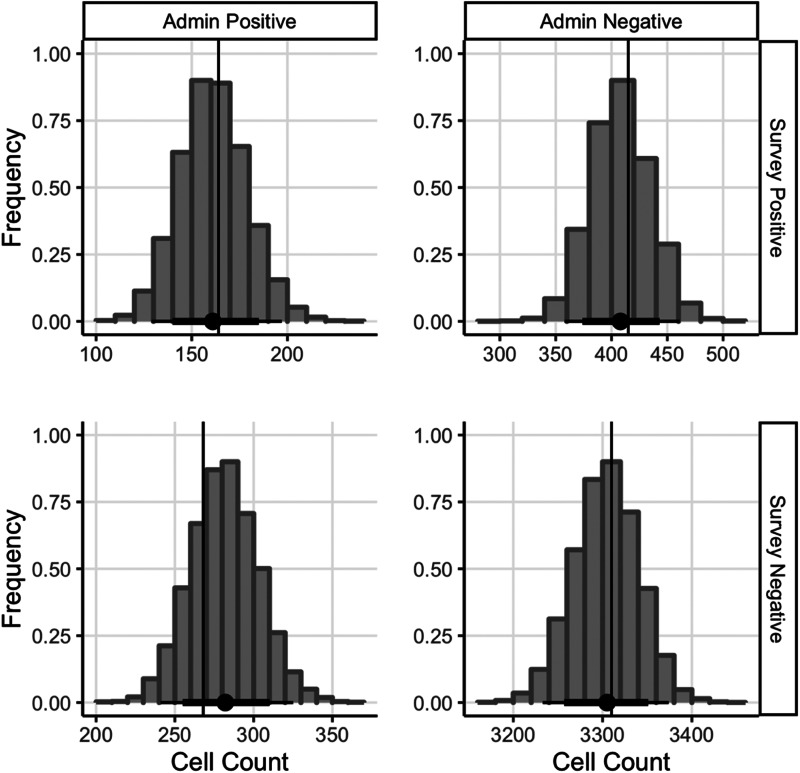
Posterior predictive checks to assess model reliability. *Note*: Our model estimates for the expected count in each cell are shown as a black dot. Associated 95% credible intervals are indicated. The vertical lines indicate the observed counts in each cell. We note that since our expectations are close to the observations, our model is capable of reproducing our data.

Stan monitors diagnostics, none of which detected problematic HMC behaviour (0 divergences, all Gelman–Rubin diagnostics <1.01, smallest effective sample size ratio was 55%). The findings from our posterior predictive checks, using simulated data (see Fig. 3), suggest that the mean of our data (*x*-axis) is similar to the mean of the posterior predictive distribution (*y*-axis), which indicates our model can reliably recreate our data (Gelman *et al*., 2013; Pananos and Lizotte, 2020).

## Discussion

We estimate that the combined prevalence of mood and anxiety disorders in Ontario, Canada, using both survey and health administrative data sources, was 8.6% (95% CI 6.8–10.6%), which sits between estimates from administrative data-derived diagnoses (mean = 7.4%) and the survey-derived diagnoses (mean = 13.9%). An in-depth discussion on the reasons why estimates from survey and health administrative data may differ can be found elsewhere (Edwards *et al*., 2019*a*, 2019*b*). Estimating the population prevalence of mood and anxiety disorders is a challenging endeavour, (Steel *et al*., 2014) and current estimates have been constrained by the properties of the measurement tools and samples. We have demonstrated how the use of a Bayesian approach may provide a more informed and accurate estimate by making use of linked survey and health administrative data, combined with prior information on the psychometric properties of these measures.

There are three reasons why we believe our combined estimate may align more closely with a true population prevalence, compared to the use of either measure alone. First, our prior work suggests that survey- and administrative-derived diagnoses may identify different sub-groups of people with a mood or anxiety disorder (Edwards *et al*., 2019*a*, 2019*b*). If both measures are identifying a discrete group of people with a spectrum of disorders at varying stages of illness and treatment, then combining both measures would provide an estimate informed by a broader distribution of the spectrum of common mental disorders in the population. Second, our estimate is the first to use prior information on established psychometric properties of the measures to inform the combined estimate. Finally, our findings align with previous research, which suggests that the true population prevalence of mood and anxiety disorders may reside between estimates derived from both measures due to the characteristics of each measure. Specifically, the depression module of the CIDI has been found to have a high false-positive rate, which may result in a falsely elevated prevalence estimate (Kurdyak and Gnam, 2005). Furthermore, compared to the estimates of depression obtained from clinical chart reviews, estimates from linked health administrative data were lower, resulting in an underestimate of the prevalence (Doktorchik *et al*., 2019). As such, it is likely that the true prevalence of mood and anxiety disorders may reside between estimates attained from the survey- and administrative-derived diagnoses, which we have demonstrated in the current study. Our findings also suggest that prior estimates of mood or anxiety disorders in Ontario, Canada using either administrative or survey data alone may be insufficient for reliably estimating a population prevalence, which has important implications for mental health policy and services.

The Bayesian approach used in this work was developed more than two decades ago (Joseph *et al*., 1995). It has been used to estimate prevalence in various clinical settings; however, forward citation searches of the seminal paper suggest there is limited use of this analytical technique for the analysis of population-level data (Joseph *et al*., 1995). Although we have been successful in adapting this approach, the increasing availability of linked data sources using multiple measures presents opportunities to build on this work going forward. Although there is a need to test the performance of this methodology in other settings with other linked measures, we believe this Bayesian approach is flexible and adaptable. The code available at GitHub provides a platform for comparing newly available linked data. Also, the ability to test model fit in Stan is a straightforward process. One potential challenge for the use of this method in other settings is deciding on priors to inform the model. This process relies on the researcher's ability to search and identify the highest quality validation studies available. We suggest the continued use of sensitivity analyses to test the robustness of the findings with variations to psychometric properties.

One of the inherent limitations of Bayesian modelling is its reliance on prior information, which in our case was the prior prevalence, concordance and psychometric estimates obtained from our linked data and external sources. As such, our analyses are limited by the accuracy of the survey- and administrative-derived diagnoses of mood and anxiety disorders. Our findings may not be generalisable to certain marginalised populations within Canada (Edwards *et al*., 2019*a*, 2019*b*), as the data limit our ability to identify some migrant groups, the homeless, institutionalised populations and Indigenous people living on reserves (Edwards *et al*., 2019*a*, 2019*b*). Furthermore, our sample may have been affected by survey non-response bias, in addition to potential bias from survey respondents who did not consent to have their data released for linkage (Louise *et al*., 2017). Also, the generalisability of the findings may be limited, as results were only derived from one province of a nationwide survey. As new data linkages become available, however, the ability to provide more granular estimates for various high-risk groups will become possible. Another limitation to this study is that prior information on the psychometric properties of the administrative data algorithm was based on depressive disorders only, which may differ from the psychometric properties for identifying anxiety disorders. This was less of a concern for our survey-derived estimates, as the psychometric properties of our measure of anxiety disorders were similar to that for depressive disorders. We used a validation study of the CIDI measuring lifetime depression, which may also have different psychometric properties than a 12-month measure. However, our sensitivity analysis evaluating the impact of a range of psychometric properties did suggest that if the true psychometric properties were different (<10%), it would not appreciably impact our combined estimate, with the exception of the specificity of our survey data measure. There has been an ongoing debate regarding the reliability and validity of structured interviews being administered by lay interviewers, as compared to clinicians, in the collection of survey data (Streiner and Cairney, 2010). We are unaware of any formal assessment of the inter-rater reliability of the interviewers in the 2012 CCHS-MH; however, the CIDI is a highly structured tool that has been shown to be reliable across many settings (Andrews and Peters, 1998).

In conclusion, accurate population-based estimates of disease are the cornerstone of health service planning and resource allocation. The current lack of a universally accepted measure of population surveillance for mood and anxiety disorders has provided an opportunity to use a unique data linkage and novel analytical techniques to improve our estimates of the prevalence of these common mental disorders. We have demonstrated how the use of Bayesian approaches may provide a more informed and accurate estimate of mood and anxiety disorders in the population. This work provides a blueprint for future population-based estimates of disease using linked health data sources.

## Data Availability

While data sharing agreements prohibit ICES from making the data set publicly available, access can be granted to those who meet pre-specified criteria for confidential access, available at http://www.ices.on.ca/DAS. The full data set creation plan is available from the authors upon request
